# The Hydrophobin Gene Family Confers a Fitness Trade-off between Spore Dispersal and Host Colonization in Penicillium expansum

**DOI:** 10.1128/mbio.02754-22

**Published:** 2022-11-14

**Authors:** Dianiris Luciano-Rosario, Justin L. Eagan, Niraj Aryal, Eddie G. Dominguez, Christina M. Hull, Nancy P. Keller

**Affiliations:** a Department of Plant Pathology, University of Wisconsin, Madison, Wisconsin, USA; b Department of Medical Microbiology and Immunology, University of Wisconsin, Madison, Wisconsin, USA; c Department of Biomolecular Chemistry, University of Wisconsin, Madison, Wisconsin, USA; Universidade de Sao Paulo

**Keywords:** Penicillium expansum, fitness, hydrophobin, tradeoff

## Abstract

Hydrophobins are small amphipathic surface proteins found exclusively in fungi. In filamentous ascomycetes, one conserved role of a subset of hydrophobins is their requirement for spore dispersal. Other contributions of these proteins to fungal biology are less clear and vary across genera. To determine the functions of hydrophobins in the biology and virulence of this fungus, we created seven single mutants and a septuple-deletion mutant (Δ*sep*) of the entire putative *P. expansum* hydrophobin gene family. One spore hydrophobin, HfbA, shared 72.56% sequence identity to the Aspergillus fumigatus spore hydrophobin RodA and was required for efficient spore dispersion in *P. expansum.* The Δ*sep* mutant was likewise reduced in spore dispersal, hypothesized to be due to the aberrant shape and clumping of the Δ*sep* conidia and conidiophores. Additionally, the Δ*sep* mutant presented several differences in physiological traits, including decreased survival in extreme cold temperatures and increased production of several toxic secondary metabolites. Most striking was the unexpected fitness advantage that the Δ*sep* strain displayed in competitive passaging with the wild-type strain on host apple where the mutant significantly increased in percentage of the colonizing population. This work uncovers potential ecological trade-offs of hydrophobin presence in filamentous fungi.

## INTRODUCTION

The hydrophobicity of filamentous fungal hyphae and spores, facilitated by amphipathic surface proteins termed hydrophobins, is an important characteristic that mediates interactions with their environment. Hydrophobins are characterized by a signal peptide sequence and eight cysteine residues that allow the proteins to form four disulfide bonds, which consequently provide hydrophobic and hydrophilic configurations to the mature protein. Once secreted, hydrophobins self-assemble into monolayers at the water-air interface of mycelia (1). Their gene copy number can vary from 2 to 40 hydrophobins in a single genome (2, 3).

Hydrophobins are required for spore dispersal in numerous species (4–6) and contribute to virulence in pathogenic fungi (7, 8). Spore-specific hydrophobins facilitate dispersal, and deletions lead to impaired spore dispersal, as has been reported for Cladosporium fulvum (4) and Aspergillus nidulans (5). In pathogenic fungi, hydrophobins mask pathogen-associated molecular patterns (PAMPs) which can be recognized by host receptors. In Aspergillus fumigatus, RodA coats conidia, forming a rodlet layer that covers the cell wall PAMPs, obscuring recognition by phagocytes (9, 10). The plant pathogen Magnaporthe oryzae produces the hydrophobin MPG1, which mediates appressorium formation and entry into host tissue (7, 11). In the Dutch Elm disease pathogen, *Ophiostoma ulmi*, the secretion of the hydrophobin cerato-ulmin is a virulence determinant that promotes symptom development (12–14).

It remains unclear why some fungi encode so many hydrophobins in their genome. For example, deletion of each hydrophobin gene in Fusarium graminearum indicated a redundancy of function in conidial formation and virulence on wheat (15). However, deletion of A. fumigatus *rodA* is sufficient to result in physical restructuring and loss of conidial hydrophobicity with no contribution by six other putative hydrophobins (16). Exhaustive analysis of hydrophobin mutants of Trichoderma guizouense and *T. harzianum* suggests that just 2 hydrophobins (out of 9 and 11, respectively) are primarily responsible for fitness traits (6). Most strikingly, the hydrophobin HFB4 played opposing roles in mediating air and water dispersal in *T. ghuizouense* and *T. harzianum.* This study showcases the intimate connection between hydrophobins and the ecology of each fungus (6).

Given the importance of hydrophobins across filamentous fungal taxa and their contributions to ecological fitness, we present our study on the full hydrophobin family (all putative hydrophobins within a genome) in the mycotoxigenic, plant-pathogenic fungus Penicillium expansum. We previously identified a putative hydrophobin (PEXP_071760, HfbC) in an RNA-profiling assessment of virulence on apple (17), leading us to hypothesize that hydrophobins could be involved in the pathogenesis of this fungus. Here, we identified seven hydrophobin-encoding genes in *P. expansum* and generated single-deletion mutants as well as a septuple-deletion mutant (Δ*sep*) to investigate their potential involvement in fungal physiology and virulence. We found that two of the single mutants, as well as the septuple mutant, exhibited decreased hydrophobicity and that one of them was severely compromised in spore dispersal. Unexpectedly, we also found that the Δ*sep* mutant showed a significant advantage in apple colonization over five successive passages in co-inoculation with the wild-type (WT) strain. These findings demonstrate that subtle, unforeseen properties of a protein class can be revealed through competitive fitness studies.

## RESULTS

### *P. expansum* encodes seven putative hydrophobins.

**FIG 1:**
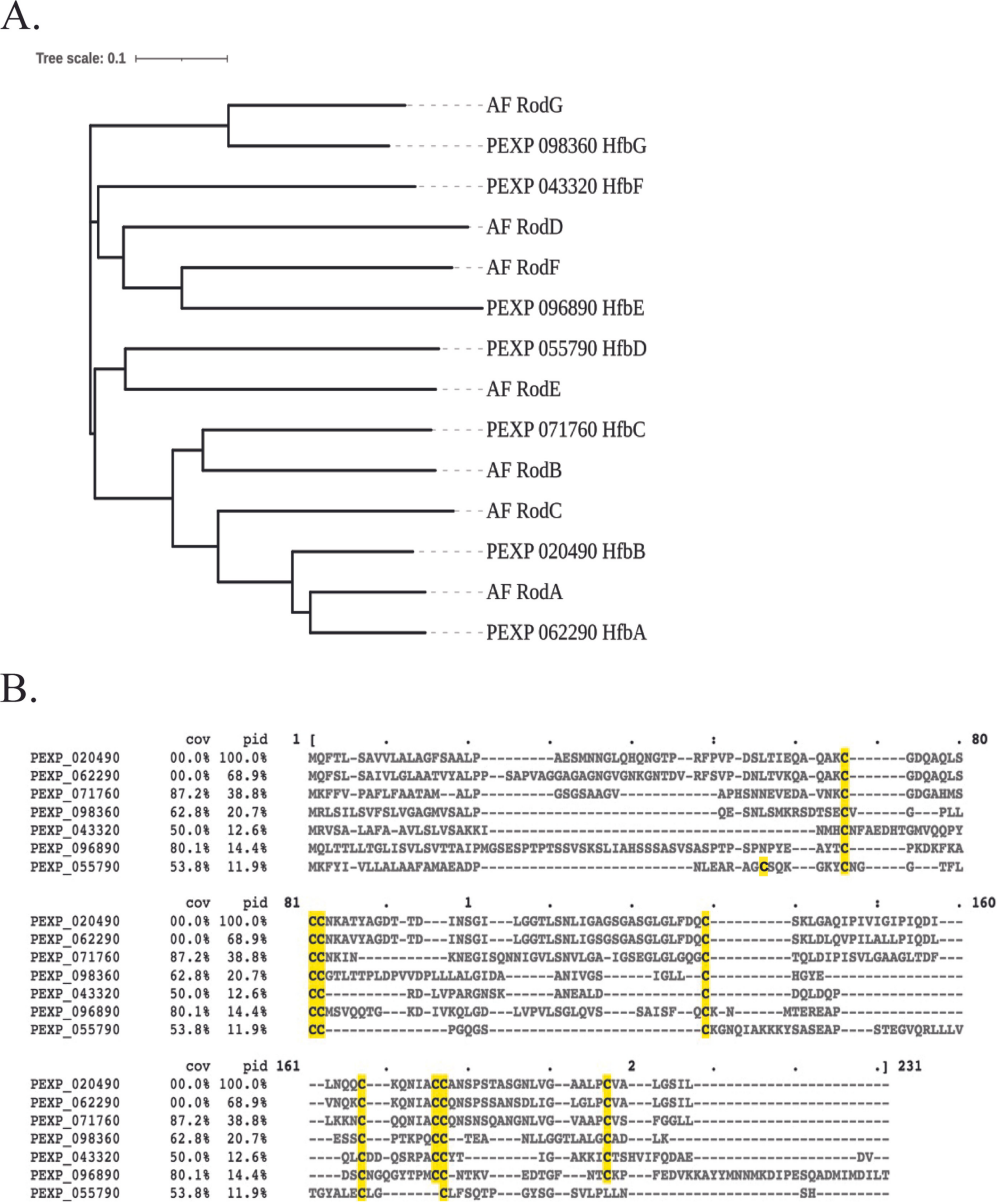
*Penicillium expansum* hydrophobin protein family. (A) Hydrophobin protein sequence dendrogram using neighbor-joining method. Multiple sequence alignment was performed using amino acid sequences for *P. expansum* and Aspergillus fumigatus hydrophobins. (B) Multiple sequence alignment of *P. expansum* hydrophobin-encoding protein sequences with cysteines highlighted.

We identified 7 hydrophobin-encoding genes (PEXP_062290, PEXP_02490, PEXP_071760, PEXP_055790, PEXP_096890, PEXP_043320, and PEXP_098360, termed *hfbA*–*G*, respectively; Fig. 1A) in the Pe21 genome using BLASTp analysis coupled with assessment of the Pe21 genome for proteins which fit hydrophobin characteristics: small size ranging from 50 to 150 amino acids (aa), signal peptide sequence, and eight cysteine residues. Out of the seven identified proteins, one (HfbD) contained only seven cysteine residues (Fig. 1B). We included this putative protein in our analysis because all of the other criteria were met, and several studies have suggested that hydrophobins contain only six or seven cysteine residues opposed to the canonical eight (2, 18).

We successfully obtained model structures for five putative hydrophobins using Phyre2. Based on sequence and structure modeling, we predict that four of these proteins (HfbA, HfbB, HfbC, and HfbE) belong to class I hydrophobins while one (HfbG) belongs to class II (Fig. S1A). We also constructed hydropathy plots, which showed the characteristic oscillating pattern similar to the known hydrophobins, predictive of the amphipathic property of these proteins (Fig. S1B). We generated a phylogenetic tree using *P. expansum* and A. fumigatus sequences because A. fumigatus also has seven hydrophobin-encoding genes (16) (Fig. 1A). Notably, HfbA shares 72.56% percent identity with the spore hydrophobin RodA, and the Phyre2 modeling results yielded a nearly identical structures of HfbA and HfbC to that of RodA (Fig. S1A).

10.1128/mbio.02754-22.3 FIG S1 Protein structure modeling and hydropathy plots of *Penicillium expansum* hydrophobins. (A) Protein structure models were generated using Phyre2 software. AF_RodA and CP_Cryparin are included for reference. Note the high similarity in the model of HfbA and HfbC to RodA. (B) Hydropathy plots were generated using Expasy Protscale. AF_RodA and CP_Cryparin are included for reference. Download FIG S1, TIF file, 2.5 MB. Copyright © 2022 Luciano-Rosario et al. 2022 Luciano-Rosario et al. https://creativecommons.org/licenses/by/4.0/ This content is distributed under the terms of the Creative Commons Attribution 4.0 International license.

### *hfbA* is expressed in spores and its loss results in decreased hydrophobicity.

**FIG 2:**
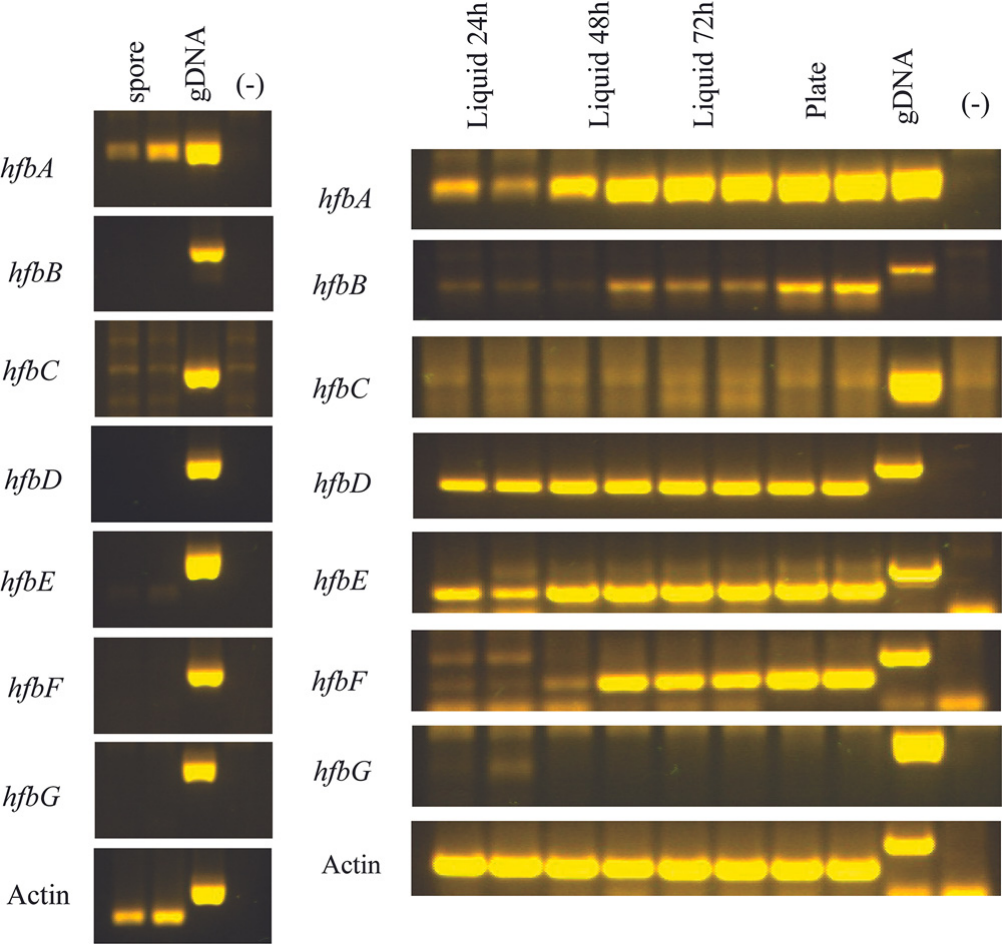
Reverse transcriptase PCR (RT-PCR) of hydrophobin-encoding genes. *P. expansum* hydrophobin genes were amplified from total cDNA synthesized from total RNA extractions from cultures grown and harvested in different conditions (spores collected from solid glucose minimal medium [GMM] 5 days postinoculation; liquid GMM cultures at 24, 48, and 72 h; and solid YES medium).

**FIG 3:**
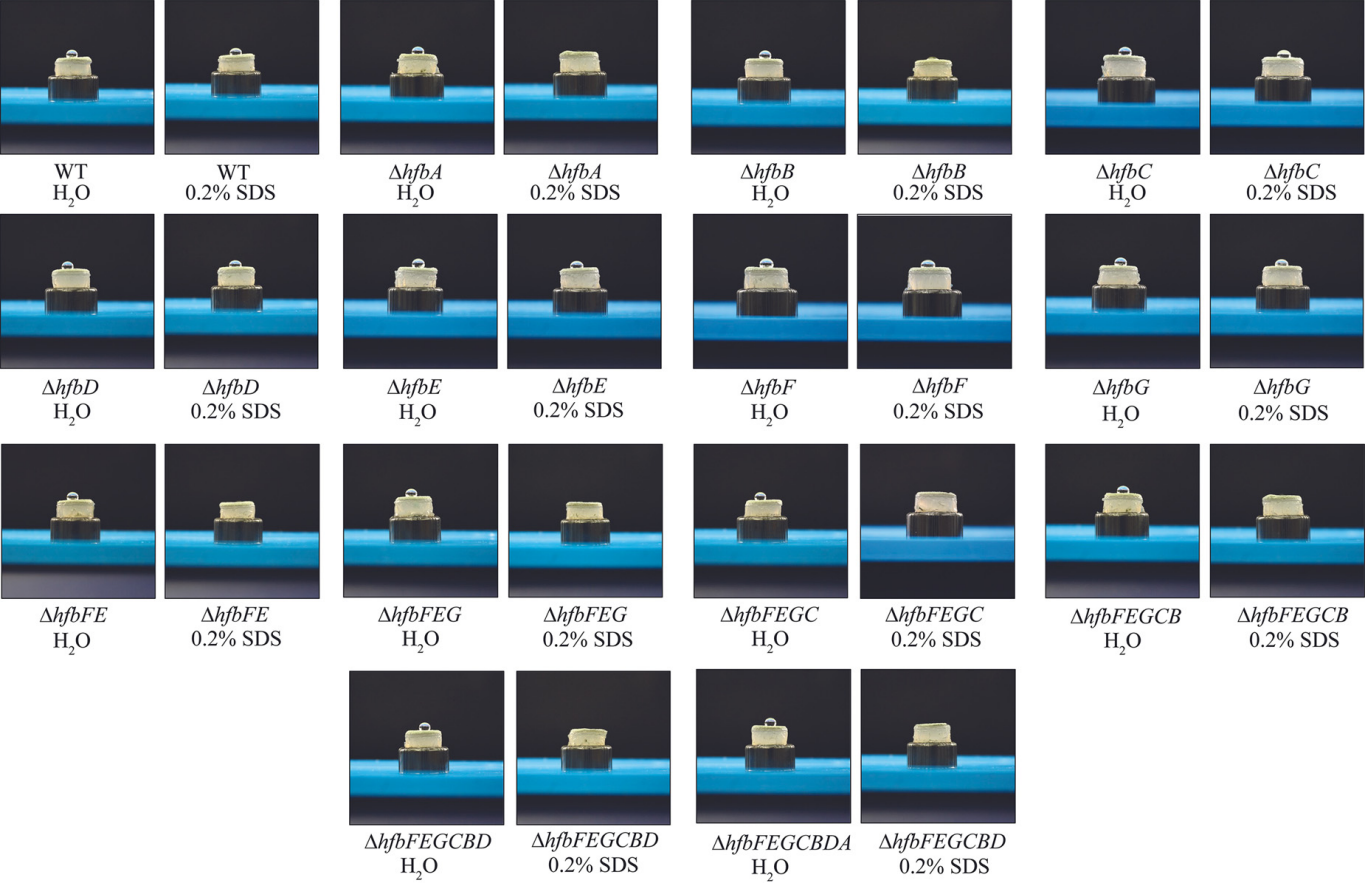
Wettability assay. *P. expansum* sporulating cultures of the wild type and a series of single through septuple hydrophobin mutant strains were imaged after adding deionized H_2_O or a 0.2% SDS 10 mM EDTA solution for 3 min.

Considering the high shared identity of HfbA with RodA, we hypothesized that *hfbA* would be expressed in the spores. We obtained expression data from six *hfb* genes, with all expressed during hyphal growth; however, *hfbA* was also uniquely expressed in spores (Fig. 2, we noticed that we could obtain amplification from unspliced *hfbF* transcript). We hypothesized that the Δ*hfbA* strain would exhibit an “easily wettable phenotype,” as described for A. fumigatus Δ*rodA* (19) and observed in hydrophobin mutants of other fungi (20) Indeed, Δ*hfbA* exhibited a wettable phenotype (Fig. 3), suggesting that the loss of a single hydrophobin is sufficient to alter *P. expansum* surface hydrophobicity.

Interestingly, we found that the loss of another single hydrophobin, HfbB, also showed the wettable phenotype. HfbB also shares high amino acid sequence similarity with A. fumigatus RodA, with 66.5% sequence similarity (compared to 72.56% similarity of HfbA). Although *hfbB* was not expressed in purified spores under our conditions, it was highly expressed during conidiophore development (Fig. 2) and may contribute to spore hydrophobicity under certain growth conditions not tested here. In analyzing the full hydrophobin family, we did not see a wettable phenotype until the loss of *hfbC–F* (Fig. 3). This suggests functional redundancy for HfbC, HfbD, HfbE, and HfbF while HfbA and HfbB are sufficient to alter fungal surface hydrophobicity as single deletants.

### HfbA mediates air and water dispersal in *P. expansum*.

**FIG 4:**
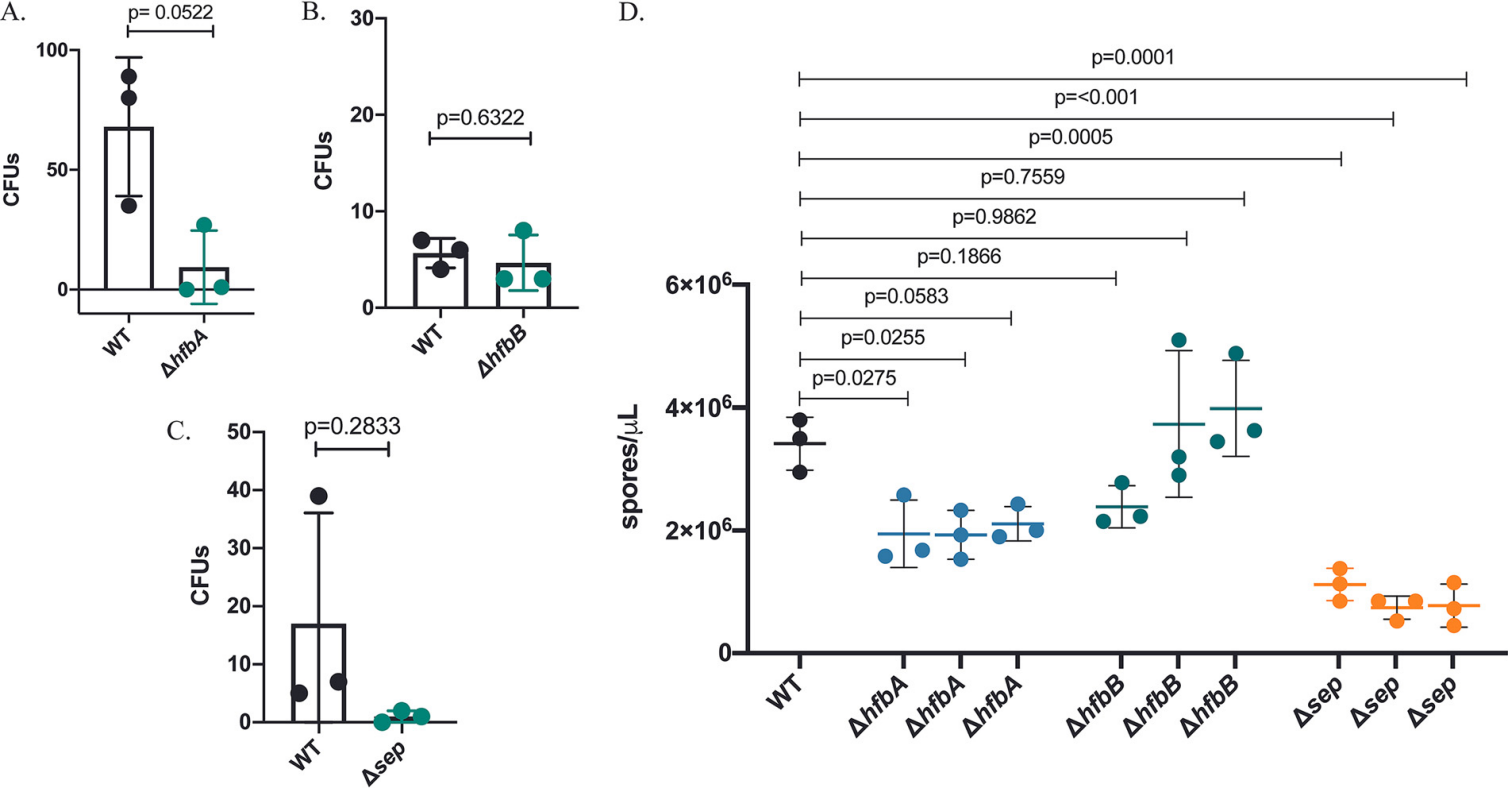
Air and water dispersal assay. Sporulating agar plugs were exposed to constant airflow for 8 s in race tubes containing GMM+UU. (A to C) Resulting CFU were quantified and plotted. (D) A 200-μL volume of water was pipetted on slanted conidiating plugs and their spores were collected and counted using a hemacytometer. Error bars indicate standard deviation across replicates. Panels A to C, Welch’s *t* test; panel D, analysis of variance with Dunnett’s multiple comparison.

RodA is required for air and water dispersal in A. fumigatus (19); thus, we hypothesized that Δ*hfbA* and possibly Δ*hfbB* would have a dispersal defect. We found that loss of *hfbA*, but not of *hfbB*, reduced air dispersal (Fig. 4A and B). Complete loss of the hydrophobin family (Δ*sep*), as expected, showed a defect similar to that of Δ*hbfA* (Fig. 4C). Consistent with these phenotypes, we found reduced water dispersal capacity for Δ*hfbA* and Δ*sep* but not for Δ*hfbB* (Fig. 4D).

**FIG 5:**
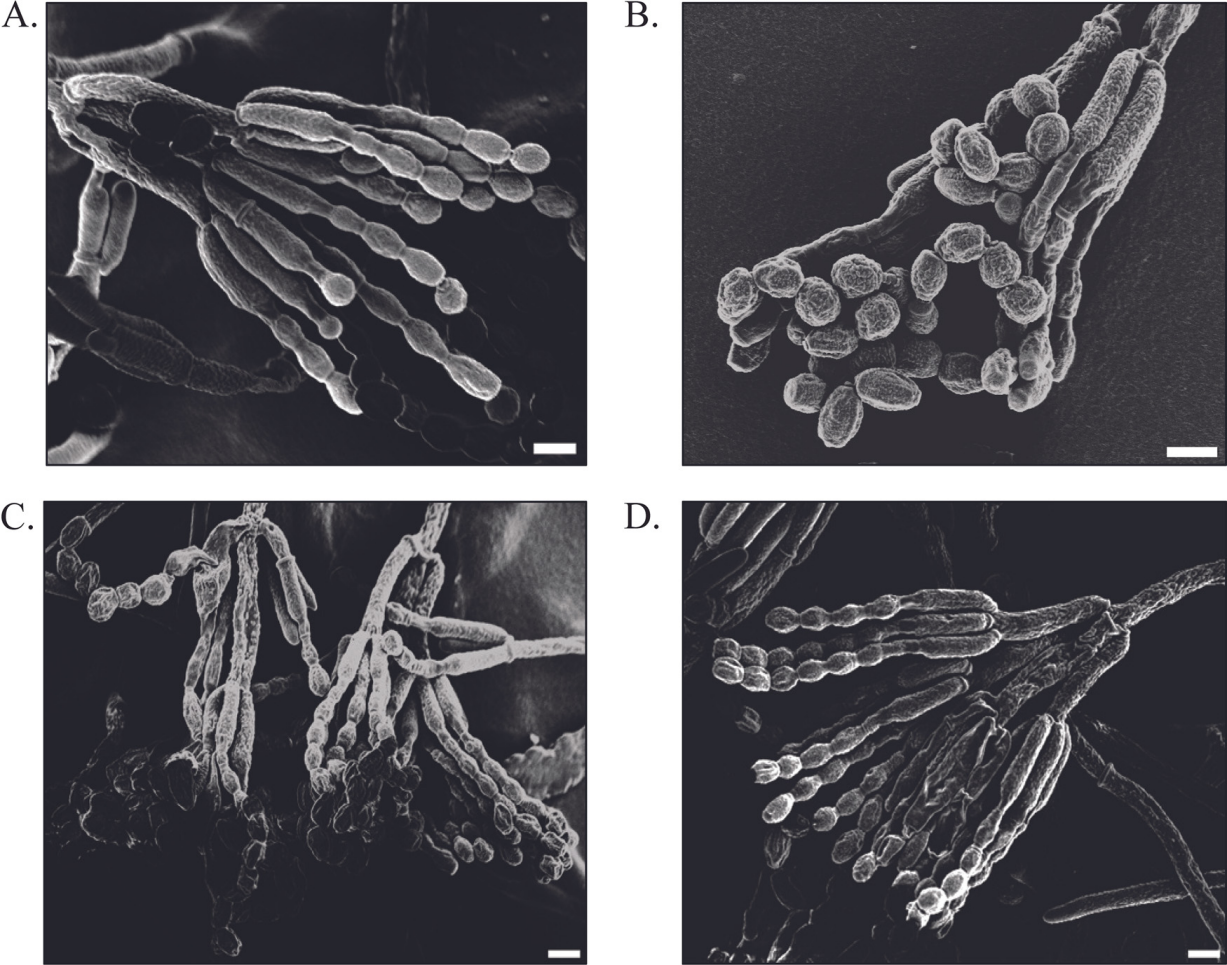
Scanning electron microscopy (SEM). Micrographs of *P. expansum* conidiophores. Representative conidiophore of (A) control strain, (B) Δ*sep* mutant, (C) Δ*hfbA*, and (D) Δ*hfbB*. Scale bars = 3 μm.

We hypothesized that dispersal defects were due to altered physical structure of spores and/or conidiophores, as has been characterized in the loss of the rodlet layer in A. fumigatus Δ*rodA* (16, 19). Scanning electron microscopy assessment of the wild type and Δ*sep* revealed strikingly aberrant conidiophore and conidial structure in the Δ*sep* strain (Fig. 5AB). The conidial chains appeared clumped together, with highly ridged conidia. We imaged Δ*hfbA* and Δ*hfbB* strains as well and found that Δ*hfbA* was the main contributor to the clumped phenotype (Fig. 5C and D). This clumped nature may contribute to the decreased dispersal properties of the mutant and its appearance is reminiscent of RodA mutants in A. nidulans (5).

Loss of the hydrophobin gene family alters germination kinetics and decreases survival under exposure to extreme cold stress.

**FIG 6:**
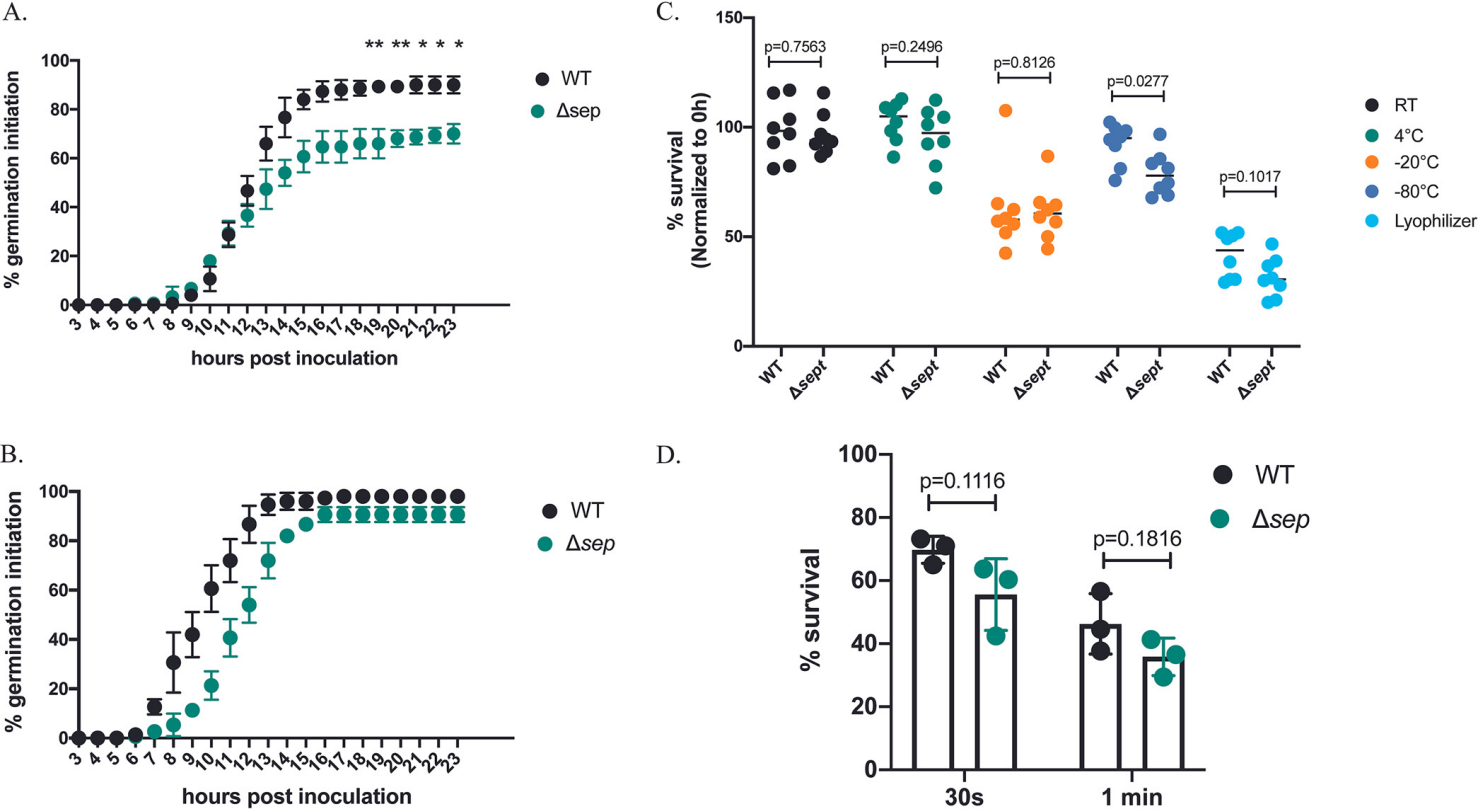
Germination assay and survival of *P. expansum* conidia subjected to different abiotic stresses. Percentage of germling formation of WT and Δ*sep* strains over 23 h in (A) GMM and (B) GMM supplemented with uridine and uracil (GMM+UU). (C) *P. expansum* spore suspensions were incubated for 25 days under different conditions (room temperature [RT], 4°C, −20°C, −80°C, and desiccation). All data were normalized to their respective controls and percent survival was calculated. (D) *P. expansum* conidia were mechanically disrupted using zirconia beads for 30 s or 1 min. Error bars indicate standard deviation across replicates. Welch’s *t* test at each time point for germination. ANOVA in panel C; multiple Student’s *t* tests in panel D.

In comparing radial growth diameters and conidia production between our wild-type control and Δ*sep*, we discovered a marker gene effect (21) where supplementation of uridine and uracil resolved these discrepancies going forward (Fig. S2). This difference was most striking in germination assays, where our Δ*sep* strain displayed modestly delayed germination in the corrected medium condition (Fig. 6A and B). All experiments subsequently used minimal medium supplemented with uridine and uracil to mitigate marker gene effect.

10.1128/mbio.02754-22.4 FIG S2 *P. expansum* deletion mutant phenotypes, radial growth assay, and conidiation assay. (A) Plates were imaged 7 days post inoculation after incubation at 25°C in glucose minimal medium supplemented with uridine and uracil (GMM+UU). Colony diameter was measured for 7 days after incubation at 25°C in (B) GMM and (C) GMM+UU. Conidia quantification in wild-type (WT) strain and Δ*sep* after culturing conidia (10^6^ spores) in (D) GMM or (E) GMM+UU. Error bars indicate standard deviation across replicates. Download FIG S2, TIF file, 2.6 MB. Copyright © 2022 Luciano-Rosario et al. 2022 Luciano-Rosario et al. https://creativecommons.org/licenses/by/4.0/ This content is distributed under the terms of the Creative Commons Attribution 4.0 International license.

Considering the aberrant structure of the conidia (Fig. 5B), we hypothesized that the Δ*sep* conidia could be susceptible to various stresses. We found no detectable reduction in spore viability between the control and Δ*sep* when incubated at room temperature, 4°C, or −20°C or in desiccation conditions. However, the mutant exhibited slightly reduced viability (~13%) when it was stored at −80°C (Fig. 6C). Spore structural integrity was assessed by subjecting conidia to mechanical stress through bead-beating. There was little, if any, difference in survival between the two strains under this regime (Fig. 6D).

Lastly, we hypothesized that our Δ*sep* strain would be more susceptible to cell wall stressors. We assessed spore viability upon exposure to common cell wall stressors (Calcofluor-white, caspofungin, and Congo red) as well as the membrane stressor sorbitol at different concentrations. There was no difference in susceptibility to any treatment between the control and Δ*sep* strains (Fig. S3).

10.1128/mbio.02754-22.5 FIG S3 Control and Δ*sep* response to cell wall stressors. *P. expansum* control and Δ*sep* were grown in the presence of various cell wall stressors: (A) Calcofluor-white (CFW), (B) caspofungin, (C) Congo red, and (D) sorbitol. Download FIG S3, TIF file, 2.5 MB. Copyright © 2022 Luciano-Rosario et al. 2022 Luciano-Rosario et al. https://creativecommons.org/licenses/by/4.0/ This content is distributed under the terms of the Creative Commons Attribution 4.0 International license.

### Loss of hydrophobin gene family results in increased mycotoxin production in media.

**FIG 7:**
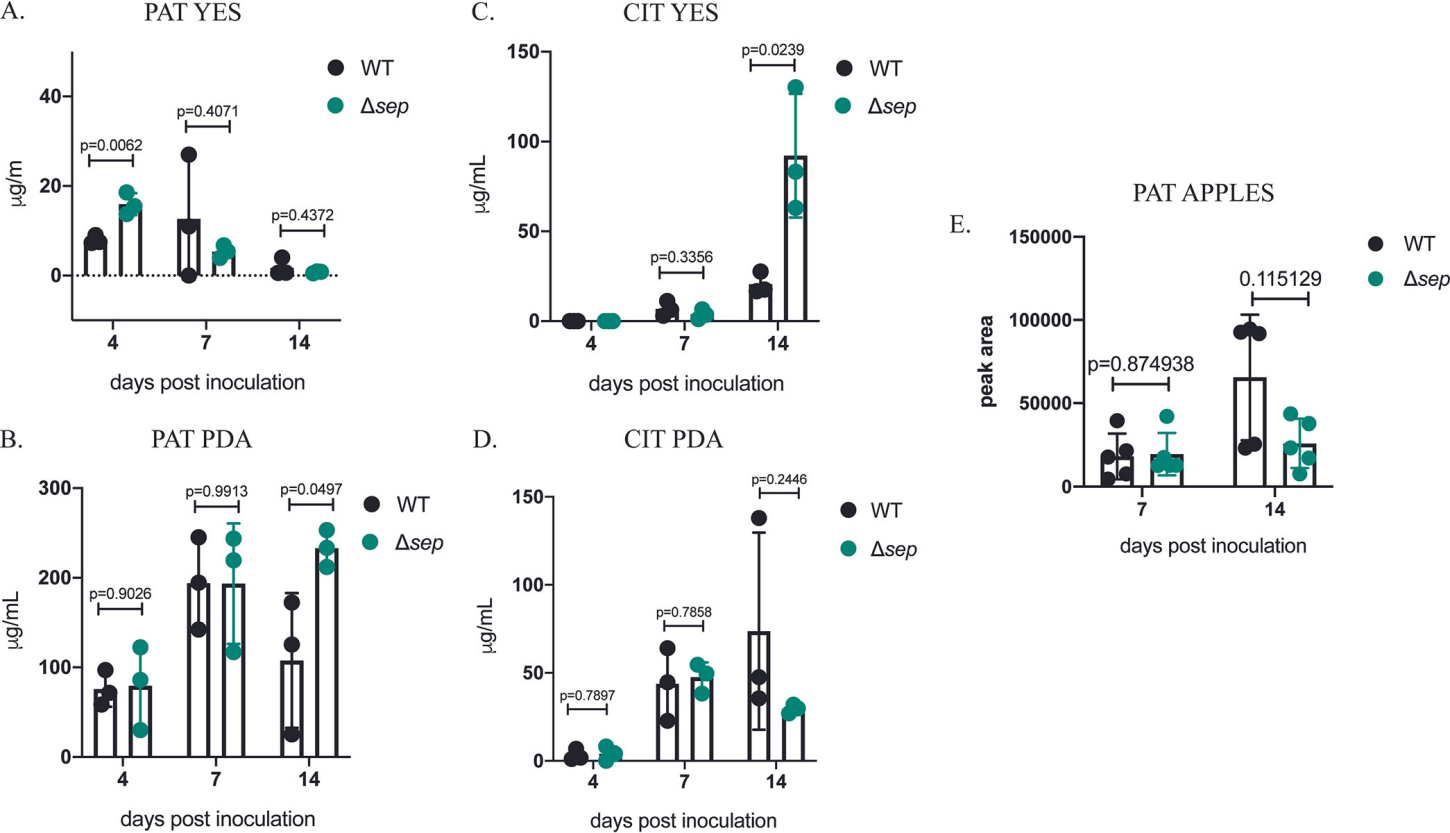
Patulin and citrinin production is increased in Δ*sep* mutant on growth media. (A and B) Patulin (PAT) and (C and D) citrinin (CIT) were quantified from metabolite extractions from potato dextrose agar (PDA) and yeast extract supplemented (YES) media after incubation at 25°C for 4, 7, and 14 days. (E) Patulin was quantified from diseased Gala apples at 7 and 14 days postinoculation. Error bars indicate standard deviation across replicates.

*P. expansum* produces two mycotoxins, patulin and citrinin (22). Thus, we sought to determine whether loss of the hydrophobin gene family impacted the production of either mycotoxin on two commonly used production media, PDA (potato dextrose agar) and YES (yeast extract supplemented) (23). As shown in Fig. 7A and B, patulin production was elevated 2-fold in YES medium at 4 days and PDA medium at 14 days. Citrinin production was ca. 4-fold higher in YES medium at 14 days (Fig. 7C) in Δ*sep* compared to that in the wild type. In contrast, citrinin was not detected in diseased apple tissue, and patulin production at 7- and 14-days postinoculation did not differ between the control and mutant (Fig. 7E).

Liquid chromatography-mass spectrometry analysis of YES medium extracts also indicated increased production of other secondary metabolites in the Δ*sep* strain, including roquefortine, communesin, and andrastatins (Fig. S4). This result follows a report in which several hydrophobin mutants of F. graminearum were found to produce higher amounts of the mycotoxin trichothecene in specific production media (Shin et al. [15]). Together, these studies suggest a previously unknown role of hydrophobins in regulating secondary metabolite production dependent on growth condition.

10.1128/mbio.02754-22.6 FIG S4 Roquefortine C, communesin A, and communesin B production are increased in *Δsep.* (A and B) Roquefortine C, (C and D) communesin A, and (E and F) communesin B were detected in metabolite extractions from potato dextrose agar (PDA) and yeast extract supplemented (YES) media after incubation at 25°C for 4, 7, and 14 days. *, *P* < 0.05; **, *P* < 0.01; ***, *P* < 0.001. Download FIG S4, TIF file, 1.0 MB. Copyright © 2022 Luciano-Rosario et al. 2022 Luciano-Rosario et al. https://creativecommons.org/licenses/by/4.0/ This content is distributed under the terms of the Creative Commons Attribution 4.0 International license.

### Δ*sep* mutant outcompetes the control strain in the apple host but not in growth media.

We hypothesized that loss of hydrophobins could result in decreased pathogenicity based on reports in other pathosystems. We assessed lesion expansion of each single hydrophobin knockout strain and of our Δ*sep* strain in the commonly used apple host “Golden Delicious,” but we found no significant reductions in disease progression compared to our control strain (Fig. S5A and C). We also assessed pathogenicity in another pome fruit host, “Bartlett” pear, and similarly detected no significant difference in virulence between the control and Δ*sep* strains (Fig. S5B).

10.1128/mbio.02754-22.7 FIG S5 Lesion development on apple and pears. A) Golden Delicious apples and B) Bartlett pears were inoculated with 10^5^ spores of WT or Δ*sep* and the lesion diameter was recorded for 7 days. C)The single and all the poly- deletion mutants were also tested in Golden Delicious apples. Error Bars indicate the standard deviation across replicates. *, *P* < 0.05; **, *P* < 0.01; ***, *P* < 0.001. Download FIG S5, TIF file, 1.3 MB. Copyright © 2022 Luciano-Rosario et al. 2022 Luciano-Rosario et al. https://creativecommons.org/licenses/by/4.0/ This content is distributed under the terms of the Creative Commons Attribution 4.0 International license.

**FIG 8:**
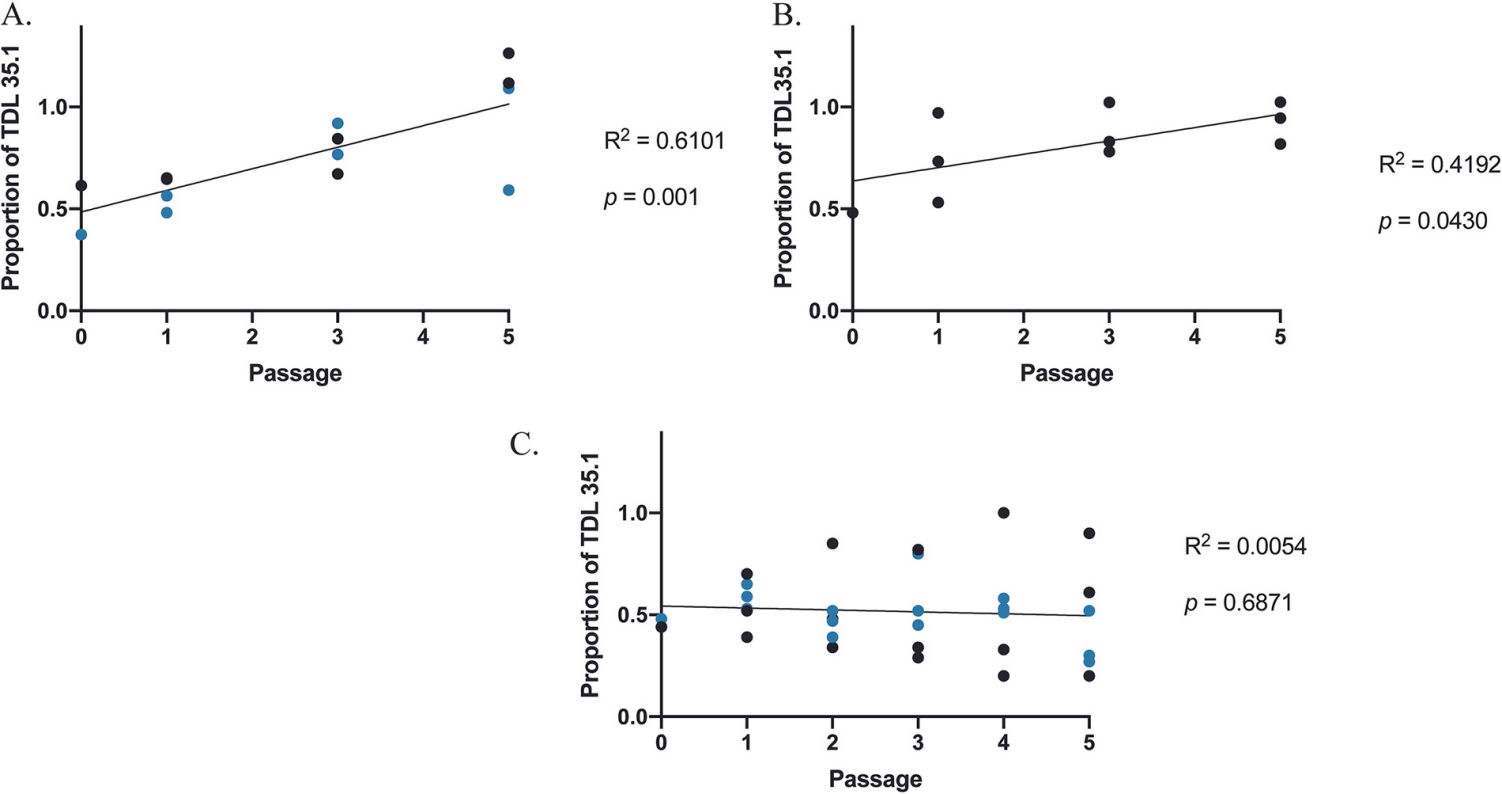
Competition assays. Passage experiments were performed in (A) Golden Delicious apples, (B) autoclaved Golden Delicious apples, and (C) GMM+UU plates. Graphs show the relative proportion of TDL 35.1 strain to the WT strain across 5 passages. Different colors represent independent experiments per panel. Line indicates simple linear regression analysis.

We considered that differences between our control and Δ*sep* strains could be subtle and not detectable by comparing lesion diameters between strains. Thus, we hypothesized that directly competing our two genotypes with equal numbers of each strain would more accurately determine fitness effects. We monitored the frequency of the Δ*sep* strain compared to the control over 5 successive disease cycles on “Golden Delicious” apples. Figure 8A shows that the Δ*sep* strain increased in relative proportion over the wild-type strain after each passage, suggesting that loss of hydrophobins resulted in increased fitness within the host environment. We next conducted a passage experiment with WT and Δ*sep* strains in autoclaved apples. We observed that the Δ*sep* also shows increased fitness in autoclaved apples (Fig. 8B) suggesting that the apple immune response did not play a measurable role in these results.

To determine whether Δ*sep* enhanced fitness was detectable independent of the host environment, we conducted the same passaging experiment *in vitro.* Interestingly, there was no significant change in the frequency of either strain at the end of the experiment (Fig. 8C). We hypothesized that the advantage Δ*sep* showed on apple could be associated with increased production of carbohydrate-degrading enzymes; however, *in vitro* assays of pectin, starch, xylan, and cellulose degradation did not show any difference between the control and Δ*sep* (Fig. S6). From these data, we conclude that deletion of the hydrophobin gene family conferred a fitness advantage to the mutant strains which only became apparent in the presence of the host tissue, although the underlying reason for this remains obscure.

10.1128/mbio.02754-22.8 FIG S6 Carbohydrate enzyme activities plate assays. WT and Δ*sep* strains were grown on (A) pectin, (B) starch, (C) xylan, or (D) cellulose as their sole carbon source. Substrate degradation was visualized using potassium iodide for pectin and starch or Congo Red for xylan and cellulose. Download FIG S6, TIF file, 0.6 MB. Copyright © 2022 Luciano-Rosario et al. 2022 Luciano-Rosario et al. https://creativecommons.org/licenses/by/4.0/ This content is distributed under the terms of the Creative Commons Attribution 4.0 International license.

## DISCUSSION

Filamentous fungal commonly contain multiple hydrophobins which bestow fitness benefits to the producing fungi. Here, we examined the role of the entire hydrophobin gene family within *P. expansum*, which unexpectedly revealed a fitness trade-off in which hydrophobins are required for spore dispersion, yet their loss increased fitness within the apple environment.

Of the seven putative hydrophobins we identified, HfbA and HfbB exhibited sequence similarity to the characterized A. fumigatus spore hydrophobin, RodA. Both single mutants displayed a loss in hydrophobicity (Fig. 3) similar to A. fumigatus Δ*rodA* (19). Interestingly, all of the polymutants also presented this phenotype, even backgrounds not containing loss of *hfbA* or *hfbB*, suggestive of functional redundancy for some of these proteins (Fig. 3). The requirement of adding SDS and EDTA into water droplets in order to observe the wettable phenotypes suggests that *P. expansum* contains other amphipathic proteins with hydrophobic properties, as found in other fungi, including cero-platanins (24), repellent proteins found in Ustilago maydis (25, 26), or CFEM (Common in several Fungal Extracellular Membrane) proteins in *Candida* species (27).

Again, similar to that in A. fumigatus Δ*rodA*, the loss of Δ*hfbA* significantly reduced dispersion in both air and water (Fig. 4A and D). Spore dispersal was not impaired in Δ*hfbB*, suggesting that HfbA alone confers spore dispersal properties in *P. expansum* (Fig. 4B and D). Concomitant with impaired dispersal, scanning electron microscopy analysis of the Δ*sep* and Δ*hfbA* mutants showed a morphological change in spores and conidiophore structure compared to the control (Fig. 5), as has been reported in studies of other fungi (5, 28). It is likely that structural changes in Δ*sep* and Δ*hfbA* spores contributed to the reduced dispersion.

Our greatest interest was assessing the fitness of the Δ*sep* mutant in host colonization because several hydrophobin mutants have been reported as being less virulent than other plant-pathogenic fungi, including F. graminearum, M. oryzae, Ophiostoma novo-ulmi, Verticillium dahliae, and Cryphonectria parasitica (7, 8, 14, 28, 29). The most powerful method for assessing the fitness of different fungal strains is through competition experiments (30–34). Therefore, we designed an *in vivo* competition passage experiment to identify any contributions that hydrophobins may provide to the progression of disease in apples. Unexpectedly, we found that the Δ*sep* strain consistently outcompeted the control strain, showing the mutant strain to be more fit than the control strain within the host environment (Fig. 8A). This fitness increase was independent of host tissue being alive or dead (Fig. 8B) but was not observed during competition on minimal growth medium (Fig. 8C).

The underlying reason(s) for this enhanced fitness remain obscure. We found no difference in mycotoxin synthesis in the mutant in apple (Fig. 7E) nor any evidence for increased carbohydrate degradation activity (Fig. S6). It is possible that the cell surface alterations of the Δ*sep* strain enhance host-derived nutrient acquisition or that the loss of hydrophobins yields an energy advantage to the mutant. Although we observed no difference in growth of this mutant on medium or apple compared to the control (Fig. S2 and S5), hydrophobin-deficient mutants of other fungi, such as V. dahliae and T. guizhouense, have shown increased growth rates on cellulose, starch, and skim milk, and on multiple carbon sources, respectively (6, 28).

Regardless of the reasons for this enhanced fitness in host tissue, our findings support an ecological trade-off hydrophobin function in *P. expansum*, where the loss of this family adversely impacts spore dispersal but enhances fitness in the host. Along the lines of our study, recent studies of hydrophobin function in the genus *Trichoderma* support potential trade-offs of different hydrophobins in spore dispersal depending on species. Loss of *hfb4* in *T. harzianum* and *T. guizhouense* generated opposite fitness costs in air dispersal. Deletion of *hfb4* in *T. harzianum* increased its aerial dispersion ability but reduced aerial dispersion in *T. guizhouense*, while showing the opposite phenotype for water dispersal (6). The most common examples of genetic trade-offs in fungi can be found in studies of antifungal resistance. For example, mutations which increased resistance to amphotericin B in Candida albicans were associated with enhanced susceptibility to various stresses, such as oxidative stress and defects in filamentation (35). Many studies have shown that fungicide resistance in plant-pathogenic fungi is almost always associated with fitness penalties and thus illustrative of evolutionary trade-offs (36). For example, in *P. expansum*, azole-resistant isolates have decreased mycotoxin production, growth, and pathogenicity (36, 37).

To better understand the evolutionary and natural history of species and determine the contribution of a trait to an organism’s fitness, we can study gene families and assess fitness-related traits at different stages of an organism’s life cycle (33). In this study, we showed that the hydrophobin gene family is essential for spore dispersal in *P. expansum* while also contributing to a fitness cost within the host environment.

## MATERIALS AND METHODS

For complete explanation of Materials and Methods, please see the supplemental methods (Text S1).

10.1128/mbio.02754-22.1 TEXT S1 Supplemental methods summary. (A) Gene expression (RT-PCR). (B) Gene deletion construct generation. (C) Protoplast isolation and transformation. (D) Hygromycin cassette excision. (E) Cell wall stressors. (F) Apple and pear pathogenicity assays. (G) Secondary metabolite analysis. (H) Plate competition experiment. (I) Carbohydrate-degrading enzyme plate assay. (J) Scanning electron microscope sample preparation and imaging. Download Text S1, DOCX file, 0.02 MB. Copyright © 2022 Luciano-Rosario et al. 2022 Luciano-Rosario et al. https://creativecommons.org/licenses/by/4.0/ This content is distributed under the terms of the Creative Commons Attribution 4.0 International license.

### Hydrophobin identification, protein modeling analysis, hydropathy plots, signal peptide prediction, and phylogenetic tree.

To identify putative hydrophobin-encoding genes, we used the BLASTp algorithm to query 51 hydrophobin protein sequences against the *P. expansum* genome. In addition, we also filtered the *P. expansum* Pe21 annotated genome using the following parameters: protein length (100 to 300 aa), number of cysteine residues (8), and NCBI annotation to meet the hydrophobin criteria. We used the default parameters of Phyre2 (http://www.sbg.bio.ic.ac.uk/phyre2/html/page.cgi?id=index) software (38) to model protein structure. Hydropathy Kyte-Doolittle plots were generated using Expasy Protscale (https://web.expasy.org/protscale/). Signal peptide predictions were made using SignalP5 (https://services.healthtech.dtu.dk/service.php?SignalP-5.0) (39). A multisequence alignment generated using the MAAFT Multiple Sequence Alignment tool from EMBL-EBI coupled with Blossum30 matrix was used to construct a phylogenetic tree (40). A Neighbor-Joining Tree was constructed using the Clustal2 program from EMBL-EBI (40). The cladogram was visualized using iTOL v6 (https://itol.embl.de/) (41).

### Background strain and culture conditions.

*P. expansum* Pe21 and mutants were used for all experiments (42). We used the strain TWW 13.1 (23) as the wild-type control. The inocula for all experiments were conidia harvested from glucose minimal medium agar plates supplemented with uridine and uracil (GMM+UU) which had been incubated at 25°C for 5 days. Conidia were harvested using a sterile 0.01% Tween 80 solution and quantified using a hemacytometer.

### Physiology: radial growth, conidiation, and germination.

GMM and GMM+UU plates were inoculated using 10^6^ spores in 7 μL and the colony radial growth was measured for 7 days. For germination rates, 5 mL liquid GMM with and without UU supplementation was inoculated with 10^5^ spores/mL, from which 1 mL was transferred to a 12-well plate. Spore suspensions were incubated at 25°C for 3 h prior to imaging for 20 h every hour. For data analysis, 50 spores per well were chosen at random and their germination was tracked for each image. In total, 3 wells per strain and 5 positions per well were assessed.

### Wettability assay.

GMM+UU plates were overlaid with molten GMM+UU inoculated with 10^6^ spores. Plates were incubated for 5 days, and then 30 μL of either deionized H_2_O or a 0.2% SDS + 50 mM EDTA solution were added to a culture agar plug for 3 min, and the wettability of the culture was observed. Photographs were taken using a Canon EOS Rebel T3 camera.

### Air dispersal assay.

GMM+UU was added and solidified in 15-cm glass race tubes. Then, a 0.5-cm overlay culture plug from a 5-day-old culture was placed on one end of the race tube. Airflow was applied to one end of the tube at 15 g/L for 8 s using an airflow meter (Dwyer Instruments, Michigan City, IN, USA). Race tubes cultures were incubated at room temperature and the resulting colonies were counted 4 days later.

### Water dispersal assay.

This protocol was modified from the methods of Cai et al. (6). Briefly, a 15° inclined plane was used with a 1 cm × 1 cm sporulating agar plug of the wild-type, Δ*sep*, Δ*hfbA*, or Δ*hfbB* strain was placed on top. Then, 200 μL of deionized water was pipetted on top of the sporulating culture and the volume was collected in a 1.5-mL tube. Spores were counted using a hemacytometer. Samples were assessed in triplicate.

### Conidia mechanical stress test.

Spore suspensions were prepared at 10^7^ spores/mL and a 500 μL suspension of the tested strains was added to a 2-mL tube containing 0.5 mL of packed 0.5-mm zirconia silica beads (Biospec, Bartlesville, OK, USA). Samples were shaken in a bead beater (Biospec MiniBeadBeater-16 Model 607, Bartlesville, OK, USA) for 0 s, 30 s, or 1 min, then diluted 1:1,000, and 20 μL was spread-plated. CFU were counted in triplicates. After this, percent survival was calculated using the following formula:

% survival= (average CFUs/average CFUs from 0−s control) ×100

This protocol was modified from the methods of Valsecchi et al. (16).

### Temporal stress test.

Conidia from the wild-type control or Δ*sep* were harvested from GMM+UU plates, spore suspensions were diluted to 2.6 × 10^8^ spores/mL, and 500 μL was aliquoted in 1.5-mL tubes. Tubes containing the spore suspensions were then incubated for 25 days in duplicate under the following conditions: room temperature (~23°C), 4°C, −20°C, −80°C, and desiccation conditions (flash-frozen and continuously lyophilized). We plated 100 spores per condition onto GMM+UU plates at time 0 and day 25. CFU were counted and percent survival was calculated and normalized to 0 h per corresponding strain. Samples were assessed using four repetitions.

### Apple competition experiment.

For the competition experiments, Δ*sep* strain TDL 35.1 (containing a hygromycin-resistance gene) was competed against the WT control. Spores were harvested from both strains independently and quantified by hemocytometer. The initial spore mixture (P_0_) was prepared in 1 mL phosphate-buffered saline (PBS) with 10^8^ spores of each strain for a total concentration of 2 × 10^8^ spores/mL. “Golden Delicious” apples were infected with 5 μL of the P_0_ spore mixture (10^6^ spores) in triplicate wound sites on each apple. Each P_0_ was used to infect two apples to incubate in parallel. After 1 week at 25°C, lesions were collected with sterilized microspatulas in 50-mL conical tubes. Lesions on one apple were pooled. Tissue was weighed and an equal volume of PBS was added to each before homogenization (Fisherbrand 150 Handheld Homogenizer Motor) for 5-s pulses, with 10 s of resting between each pulse, until the tissue was able to be pipetted. New apples were surface-sterilized and wounded before being infected with 5 μL of the homogenized tissue from the previous passage. Each tissue homogenate was stored in 25% glycerol as −80°C stocks. To enumerate the relative ratios of each strain, glycerol stocks of P_0_ mixtures were thawed at room temperature and diluted to 10^3^ spores/mL to be spread-plated at 100 μL onto 10 GMM + ampicillin [100 μg/mL] and 10 GMM + hygromycin [100 μg/mL] plates. CFU were enumerated after 3 days of incubation at 25°C. For apple passages P_1_, P_3_, and P_5_, each tissue homogenate was thawed at room temperature and diluted 1:100 in PBS before spread-plating on 5 GMM + ampicillin (100 μg/mL) and 5 GMM + hygromycin (100 μg/mL) plates before CFU were quantified after 3 days at 25°C. This experiment was conducted in duplicate and repeated twice using the Δ*sep* strain and once with the Δ*hfbFEGCB* mutant.

The competition assay on autoclaved apples was performed similarly, with minor modifications. The initial P_0_ spore mixture was created as above. For each passage, three “Golden Delicious” apples were autoclaved (15-minute sterilization, slow exhaust) in glass beakers and cooled to room temperature before wounding. Apples were wounded at three sites per apple along the perimeter using a sterile toothpick and inoculated with 5 μL of the P_0_ mixture. After 1 week at 25°C, each apple in its entirety was homogenized using a hand blender within a glass beaker containing 200 mL PBS. The apples for the next passage were infected with 5 μL of the tissue homogenate. Each passage was stored as 25% glycerol stocks to store at −80°C. To determine the relative ratios of each strain, the tissue homogenates were thawed completely and diluted sufficiently in PBS to plate onto 5 GMM + tetracycline (20 μg/mL) and 5 GMM + hygromycin (100 μg/mL). The initial ratio within the P_0_ mixture was determined by plating onto 10 of each antibiotic plate. A simple linear regression was used as a statistical test using GraphPad Prism version 8.

### Statistical analysis.

All statistical analyses were performed using Graph Pad Software Prism version 8. Data are reported as means and standard deviation. Welch’s *t* test was performed to determine statistical differences between strains for pairwise comparisons, and analysis of variance with multiple comparisons was used to compare different treatments. For the competition study, data from independent experiments were pooled and a simple linear regression was employed.

10.1128/mbio.02754-22.2 TABLE S1 (A) List of primers used in construct generation and (B) reverse transcriptase PCR. (C) *Penicillium expansum* strains used in this study and their genotypes. Download Table S1, DOCX file, 0.03 MB. Copyright © 2022 Luciano-Rosario et al. 2022 Luciano-Rosario et al. https://creativecommons.org/licenses/by/4.0/ This content is distributed under the terms of the Creative Commons Attribution 4.0 International license.

10.1128/mbio.02754-22.9 FIG S7 Southern blot analyses confirm correct constructs of deletion mutants. Each panel (A to M) contains a schematic of the DNA digestion design, expected size, resulting strain, and blot image. Download FIG S7, TIF file, 2.8 MB. Copyright © 2022 Luciano-Rosario et al. 2022 Luciano-Rosario et al. https://creativecommons.org/licenses/by/4.0/ This content is distributed under the terms of the Creative Commons Attribution 4.0 International license.
