# Supplementary material for: The Hydrophobin Gene Family Confers a Fitness Trade-off between Spore Dispersal and Host Colonization in Penicillium expansum
Source: mBio. 2022 Nov 14;13(6):e02754-22. doi: 10.1128/mbio.02754-22 (PMC9765440; doi:10.1128/mbio.02754-22)
Supplement: TEXT S1 [file mbio.02754-22-s0001.docx]

**Supplemental Methods**

**Supplemental Methods Summary:**

1. Gene Expression (RT-PCR)
2. Gene Deletion Construct Generation
3. Protoplast isolation and transformation
4. Hygromycin Cassette Excision
5. Cell Wall Stressors
6. Apple and Pear Pathogenicity Assays
7. Secondary Metabolite Analysis
8. Plate Competition Experiment
9. Carbohydrate degrading enzyme plate assay
10. Scanning Electron Microscope Sample Preparation and Imaging

Gene Expression (RT-PCR)

The *P. expansum* wild type Pe21 strain was cultured in different conditions, and total RNA was extracted. Fifty mL liquid GMM were inoculated with 10^6^ spores/mL and incubated for 24, 48, and 72 hours at 25°C with shaking at 250 rpm. Also, point inoculations were grown in YES media as *P. expansum* grows mostly mycelia in this media and spores were harvested from GMM plates 5 days post inoculation as GMM is optimal to obtain conidia. Resulting cultures were harvested and lyophilized, and approximately 0.1 g of lyophilized cell mass was macerated in a 1.5 mL tube using a spatula. After that, 1 mL of QIazol Lysis Reagent (Qiagen, Hilden, Germany) reagent was added to the pulverized tissue and mixed by inversion. The sample was extracted with 250 μL of chloroform for 5 minutes at room temperature and centrifuged for 15 minutes at 12,000 rpm at 4°C. The supernatant was then extracted with an equal volume of phenol:chloroform:isoamyl alcohol (25:24:1). Samples were mixed by inversion, incubated at room temperature for 5 minutes and centrifuged for 15 minutes at 12,000 rpm. The supernatant was then mixed with 750 μL of 100% ethanol followed by a 10 minute incubation at room temperature. Samples were centrifuged at 12,000 rpm for 10 minutes at 4°C, and the resulting pellet was washed with 1 mL of 75% ethanol and dried at room temperature for approximately 15 minutes. The dried pellet was resuspended in 40 μL of diethyl pyrocarbonate (DEPC)-treated water and incubated in a water bath at 65°C for 30 minutes. To assess RNA quality and quantity, each sample was visualized on an agarose gel and assessed using an Epoch 2 Microplate Spectrophotometer. For cDNA synthesis, 1 μg of total RNA was converted to cDNA using Bio-Rad iScript cDNA Synthesis Kit according to the manufacturer’s instructions. For assessing gene expression of the *P. expansum* hydrophobin-encoding genes using RT-PCR, primers were designed flanking an intron to distinguish amplification from genomic DNA (gDNA) or complementary DNA (cDNA) (Table S1B). The PCR was performed in 25 μL reactions containing 1X GoTaq Buffer, 0.5 μL of 10 mM dNTPs (Promega, Madison, WI, USA), 0.25 μL of 100 μM primer forward and reverse, 1 μL of in-house DNA taq polymerase, 1 μL of 1:5 diluted cDNA, and 17 μL of sterile deionized H_2_O. Cycle conditions used were 94°C for 5 minutes, 36 cycles of 95°C for 30s, 60°C for 30s, and 72°C for 30s and a final incubation for 1 minute at 72°C. Products were visualized using 2% agarose gel electrophoresis.

Gene Deletion Construct Generation

Double joint PCR was used to generate the transformation cassettes as described by Lim *et al.*, 2012. Briefly, for each gene, the 1.5 kb regions upstream and downstream of the open reading frame (ORF) and the selectable marker genes were amplified using Pfu DNA polymerase (Agilent, Santa Clara, CA, United States). The primers contained a 30 bp overhang complementary to the selectable marker sequence which flanked it. The selectable markers used were *A. fumigatus* *pyrG* for the single deletion mutants and an excisable hygromycin-conferring resistance cassette from the plasmid psk529 (Hartmann *et al.*, 2010; Jiménez-Ortigosa *et al.*, 2012) for the polymutant strains. After obtaining each flank individually, these products were combined in a 1:3:1 ratio (5’ flank: selectable marker: flank:3’ flank) and PCR-amplified with primers to the distal ends of the flanks. The resulting products purified using a G-50 column. The primer sequences for construct generation are listed on Table S1A.

Protoplast isolation and transformation

Gene replacement by homologous recombination and protoplast-mediated transformation was used to obtain the deletion mutants as previously described by Greco *et al.*, 2019 with some modifications. For the single deletion mutants, the background strain used was TWW12.1 (Wang *et al.*, 2021). For the polymutant, the background strain was TDL 17.2 (Δ*hfbF*). To generate protoplasts, 10^9^ spores were incubated at 25°C in liquid GMM 0.002% Yeast Extract with shaking at 280 rpm until germlings were visible. Germlings were recovered via centrifugation and washed in water. Approximately 0.5 g of the resulting pellet was suspended in 30 mL of protoplasting solution (Osmotic Media, 0.06 g Yatalase, 0.1 g Lysing Enzymes from *Trichoderma*) and incubated for four hours at 28°C and shaking at 100 rpm. Resulting protoplasts were isolated via centrifugation in 10 mL of Trapping Buffer for 15 minutes at 1,500 rpm. The protoplasts were washed with STC Buffer (1.2M sorbitol, 10mM CaCl_2_, 10mM Tris-HCl, pH 7.5) and resuspended in a final volume of 250 μL STC. For transformation, 100 μL of protoplasts were incubated with 20 μL of the appropriate construct in a final volume of 200 μL of STC solution on ice for 50 minutes. Then 1.5 mL of a 60% PEG Solution were added and mixed gently by inversion. A 15-minute incubation at room temperature was then followed by the addition of 5 mL of STC buffer. The resulting solution was gently mixed by inversion in 5 mL molten SMM top agar media and plated on SMM medium with the appropriate supplements. After 5 days, the resulting colonies were isolated, their DNA extracted, and the DNA was screened by PCR and Southern blot to identify transformants with integration of the construct into the proper locus and no ectopic integration events (Fig. S8).

Hygromycin Cassette Excision

The selectable marker cassette used for generating the polymutant deletion strains, contains a β-recombinase enzyme driven by a xylose promoter (Hartmann *et al.*, 2010; Jiménez-Ortigosa *et al.*, 2012). When grown in 2% xylose in the absence of glucose, the β-recombinase excises the selectable marker cassette. To re-use hygromycin as a selectable marker for construction of the polymutant strains, hygromycin-resistant strains were grown on xylose minimal media and then tested for susceptibility to 100 μg/mL hygromycin. Susceptible strains were used as the background strains for the subsequent gene deletions. A list of the strains used in this study are listed in Table S1C.

Cell Wall Stressors

GMM+UU was prepared containing Congo Red (CR), Calcofluor White (CFW), Caspofungin, or Sorbitol. Final concentrations of 25μg/mL, 50μg/mL, 75μg/mL, 100μg/mL, and 200μg/mL were tested for CFW and 10μg/mL, 25μg/mL, 50μg/mL, 100μg/mL, 150μg/mL, and 200μg/mL for CR, and 0.125μg/mL, 0.25μg/mL, 0.5μg/mL, 1μg/mL, and 8μg/mL for Caspofungin. For sorbitol stress, plates contained sorbitol to obtain a final concentration of 1M, 1.2M or 2M. These were compared to a non amended GMM+UU or GMM+UU+DMSO control plate as the Caspofungin stock solution was prepared with DMSO (dimethyl sulfoxide) as a solvent.

Apple and Pear Pathogenicity Assays

“Golden Delicious” apples and “Bartlett” pears were surface sterilized using 2% sodium hypochlorite for 2 minutes. The fruits were then rinsed with sterile deionized water. Lesions were generated using sterile toothpicks and creating a wound 0.5 cm deep. After this, apples and pears were incubated at room temperature for 15 minutes and then inoculated with 7 μL containing 10^5^ spores. This assay was also performed using 10^3^ spores for the WT and Δ*sep* strains. Four wounds were generated per apple and each apple was inoculated with the control and mutant strain of interest in order to account for apple to apple variation. For pears, due to size limitations, one wound per pear was generated. Lesion diameters were measured for 7 days after inoculation. Five replicates were used.

Secondary Metabolite Analysis

WT and Δ*sep* mutant were grown in solid and liquid cultures at 25°C using the mycotoxin production media YES (0.005% yeast extract and 3% glucose) and PDA (potato dextrose agar). Cultures were grown in triplicates for 4, 7 and 14 days in YES and PDA media and were then extracted for secondary metabolite analysis. Whole agar plates from YES and PDA plates were cut into small pieces using a spatula and placed in a 50 mL tube. Then, 25 mL of methanol were added to the vial, and samples were sonicated for 30 min. Samples were vortexed and filtered via Whatmann filter paper (11 cm). Samples were left in fumehood overnight for drying. Dried crude extracts were resuspended in 2 mL of 50:50 methanol: water and filtered using 0.2-mm Target2 polytetrafluoroethylene (PTFE) syringe filters (Thermo Fisher). Ten-microliter volumes of samples were subjected to ultrahigh-performance liquid chromatography coupled with high-resolution mass spectrometry (UPLC-HRMS), which was performed on a Thermo Scientific-Vanquish UPLC system connected to a Thermo Scientific Q-Exactive Orbitrap mass spectrometer in ES1 and ES2 modes between 100 *m/z* and 1,800 *m/z* to identify metabolites. Acquity C_18_ column (100 x 2.1 mm, 1.7 µm 1000 Å) was used with 0.05% formic acid in acetonitrile (organic phase) and 0.05% formic acid in water (aqueous phase) as solvents at a flow rate of 0.2 ml/min. A solvent gradient scheme was used, starting at 20% organic for 2 min, followed by a linear increase to 98% organic over 13 min, holding at 98% for 5 min, decreasing back to 20% organic for 1 min, and holding at 20% organic for the final 4 min, for a total of 25 min. Data acquisition and procession for the UPLC-MS were controlled by using Thermo Scientific Xcalibur software. For comparative metabolomics analysis, ThermoRAW mass spectral profiles were converted to mzML format using Proteowizard^1^ following which they were processed using Mzmine 2.53^2^. The MS1 data, represented as a peak list containing m/z values, retention times, and peak areas for each feature across fungal samples, was exported as a .csv file. Peak area was extracted from the file to plot the comparative bar graph (if the graph were done in supplementary). For volcano plots, mzml data were imported to XCMS, an open-source package (<https://xcmsonline.scripps.edu/>). Differential masses found via XCMS were filtered by having a maximum intensity greater than 1E+07. Identified masses having a maximum intensity lower than 1E+07 were considered as a background. Secondary metabolites annotations were done based on our previous work by Tannous *et al.*, 2018.

To quantify the production of citrinin in control strain and septuple mutant strain, a standard curve was built using a series of diluted (from 0.001 mg/ml to 1 mg/ml) citrinin standards (Cayman Chemical) by high-performance liquid chromatography (HPLC; Gilson) with a 10-ml injection. A YMC-Pack ODS-A column (4.6 x 250 mm, 5 µm particle size, pore size120 Å) was used with 0.1% formic acid in acetonitrile (organic phase) and 0.1% formic acid in water (aqueous phase) as solvents at a flow rate of 0.8 ml/min. A solvent gradient scheme was used,
starting at 30% organic, followed by a linear increase to 100% organic over 10 min, holding at
100% for 5 min and decreasing back to 30% organic within a total of 25 min. The standard curve was built linking the concentration and the peak area at 330 nm and was formulated as y = 24,583x with an R^2^ of 0.9515. The concentration of citrinin in different strains was then calculated based on the standard curve. Patulin quantification was done in the similar way by monitoring peak area at 276 nm and was formulated as y = 111574x with an R^2^ of 0.9993.

For mycotoxin quantification in apple, 5g of tissue from inoculation site were sliced. Subsequently, 25 ml of methanol were poured in sterile falcon tube containing apple tissues. This was followed by vortexing (1min) and solvent was filtered out via whatmann filter paper. Dried extracts were then resuspended in 2 ml of 50:50 (MeOH:Water). Finally, sample was filtered via PTFE filter before HPLC injection.

Plate Competition Experiment

Similarly to the apple competition assay, spores were harvested from WT and Δ*sep* mutant grown on GMM+UU independently before quantifying spore concentrations via hemocytometer counts. Spore suspensions were diluted and combined in equal numbers in order to obtain 2 X 10^6^ spores in 8 μL. This mixture was then point inoculated in GMM plates in triplicate and every 7 days, the spores were harvested, counted and 2 X 10^6^ spores were point inoculated to new GMM plates independently. Each week, 150 spores from the spore suspensions were spread plated in GMM and GMM supplemented with Hygromycin at 100μg/mL containing plates in order to quantify CFUs and calculate the strains ratio as the WT strain has no hygromycin resistance while the *Δsep* mutant (TDL 35.1) has a hygromycin resistance conferring gene. This experiment was conducted in triplicate and repeated two times. A simple linear regression was used as a statistical test using GraphPad Prism version 8.

Carbohydrate degrading enzyme plate assay

Minimal media plates were prepared using 1% starch, 1% pectin, 0.5% xylan, and 1% carboxymethyl cellulose (CMC) as a sole carbon source. Plates were inoculated with 10^6^ spores and after 72 hours, hydrolysis zones were visualized by flooding the plates with a 4% potassium iodine/ 1% iodide solution (starch and pectin) or 0.1% Congo Red (xylan and cellulose). Plates were incubated for 15 minutes before imaging.

Scanning Electron Microscope Sample Preparation and Imaging

*P. expansum* WT, Δ*hfbA*, Δ*hfbB*, and Δ*sep* colonies were grown over 10 mm Whatman paper disks embedded approximately 1-2 mm below the surface of GMM+UU plates. Briefly, 6 µL of an inoculum of 10^6^ spores cultured were placed directly over the center of Whatman disks embedded below the surface of the agar and incubated at 25°C. After 72 hours, sterile forceps were used to remove the Whatman disks containing the samples and were placed into 12 well plates. Then, 1 ml of fixative (4% formaldehyde, 1% glutaraldehyde in PBS) was added to each sample and incubated overnight at room temperature. Whatman disks were then washed with PBS and treated with 1% osmium tetroxide for 30 min at ambient temperature. After a series of alcohol washes (30 to 100%), final desiccation was performed by critical-point drying. Whatman disks were mounted, palladium – gold coated, and imaged in a scanning electron microscope (Zeiss Gemini SEM 450) at 3 kV. The images were assembled using Adobe Photoshop 23.3.0.
