# Supplementary material for: The Hydrophobin Gene Family Confers a Fitness Trade-off between Spore Dispersal and Host Colonization in Penicillium expansum
Source: mBio. 2022 Nov 14;13(6):e02754-22. doi: 10.1128/mbio.02754-22 (PMC9765440; doi:10.1128/mbio.02754-22)
Supplement: TABLE S1 [file mbio.02754-22-s0002.docx]

| Table S1A**. List of primers used for construct generation** | | |  |
| --- | --- | --- | --- |
| **Strain** | **Product** | **Primer** | **Sequence** |
|  | 5' flank | DL_Pexp_062290_5_F | CAC CCT CAC TGT TTT AGT GGG ACG |
|  |  | DL_Pexp_062290_5_R | CAC AGT GGA GGA CAT ACC CGT AAT TTT CTG TGT GAT GGT TTG GAT TTG AAC GAG AGG |
|  | pyrG | DL_Pexp_AfumiPyrG_F | CAG AAA ATT ACG GGT ATG TCC TCC AC |
|  |  | DL_Pexp_AfumiPyrG_R | TCA TGA CTT GCC GCA TAC TCT GG |
|  | 3' flank | DL_Pexp_062290_3_F | TAT ATG GCC AGA GTA TGC GGC AAG TCA TGA TTA GAT GTC CTG TTC ATT TCA TAT CTC GTG |
| ∆*hfbA* (PEXP_062290) |  | DL_Pexp_062290_3_R | TAT CTC CAG CCC AGA ATT GAA TGC |
|  | construct | DL_Pexp_062290_5N_F | GTA ATG CGG ACT ATG TAT AGA CCA AGG |
|  |  | DL_Pexp_062290_3N_R | GAT GCC AAT ATA CCC TCC AAC CAG C |
|  | confirmation | DL_Pexp_062290_ORF_F | GAC CAG GCT CAG CTC TCT TGC |
|  |  | DL_Pexp_062290_ORF_R | GGA GTT CTG TGA TGT GTT AGT TGA GC |
|  |  |  |  |
|  | 5' flank | DL_PEXP_020490_5F | GCA TGT TGG TGG AAT AAT GTC TGT GG |
|  |  | DL_PEXP_020490_5R | ACA GTG GAG GAC ATA CCC GTA ATT TTC TGC TTG AAG GTA TGG TAT TGA AAG ATT AAA ATC |
|  | pyrG | DL_Pexp_AfumiPyrG_F | CAG AAA ATT ACG GGT ATG TCC TCC AC |
|  |  | DL_Pexp_AfumiPyrG_R | TCA TGA CTT GCC GCA TAC TCT GG |
|  | 3' flank | DL_PEXP_020490_3F | TAT ATG GCC AGA GTA TGC GGC AAG TCA TGA GCT CAT CGC TTT GCA GTC AGT C |
| ∆*hfbB* (PEXP_020490) |  | DL_PEXP_020490_3_R | TAC TTA GTC GCC CGT ATA TGT AGC |
|  | construct | DL_PEXP_020490_N_F | ACA CTA ACA TCC ACT TCC TGT GAA GC |
|  |  | DL_PEXP_020490_N_R | AGG ATT CAC CAA CTG GAC TTT CAC G |
|  | confirmation | DL_PEXP_020490_ORF_F | ATG CAG TTC ACT CTC TCC GCC |
|  |  | DL_PEXP_020490_ORF_R | GCG TTG AAA GGT GAT GGG TGC |
|  |  |  |  |
|  | 5' flank | DL_PEXP_071760_5_F | TCG GCT GTG CCT TAT ATT CAA GAC C |
|  |  | DL_PEXP_071760_5_R | CAC AGT GGA GGA CAT ACC CGT AAT TTT CTG TTT GAT AGA GGC TTT AGA GAA GGC GG |
|  | pyrG | DL_Pexp_AfumiPyrG_F | CAG AAA ATT ACG GGT ATG TCC TCC AC |
|  |  | DL_Pexp_AfumiPyrG_R | TCA TGA CTT GCC GCA TAC TCT GG |
|  | 3' flank | DL_PEXP_071760_3'_F | TTA TAT GGC CAG AGT ATG CGG CAA GTC ATG AAT TGC ATT TCA TTG AGT GCA GCC TTT TCC |
| ∆*hfbC* (PEXP_071760) |  | DL_PEXP_071760_3_R | GTA TCA AGG GAA CCG TAA AGC TTC C |
|  | construct | DL_PEXP_071760_N_F | GGA TAT TCG ACG CCA TGC ATA TAC TGA |
|  |  | DL_PEXP_071760_N_R | TGG GGT TTA TTC AGG TTC TTC TCG C |
|  | confirmation | DL_PEXP_071760_ORF_F | CTT TTC GCT GCT ACT GCC ATG G |
|  |  | DL_PEXP_071760_ORF_R | ATC ATT TTG GGG GAG CAC ATA CAG C |
|  |  |  |  |
|  | 5' flank | DL_PEXP_055790_5_F | TCT ATA AAA AGC CCA ATG CCC ACG C |
|  |  | DL_Pexp_055790_5_R | AGT GGA GGA CAT ACC CGT AAT TTT CTG GGT TGA TTG ATT TGA AAA GAT TGT TCA AAG TTG |
|  | pyrG | DL_Pexp_AfumiPyrG_F | CAG AAA ATT ACG GGT ATG TCC TCC AC |
|  |  | DL_Pexp_AfumiPyrG_R | TCA TGA CTT GCC GCA TAC TCT GG |
|  | 3' flank | DL_Pexp_055790_3_F | TAT ATG GCC AGA GTA TGC GGC AAG TCA TGA TGA AAA TGT CCA TCT GCA ATG AAA GCC |
| ∆*hfbD* (PEXP_055790) |  | DL_Pexp_055790_3_R | ATC GTT GTT GTC CGG AGG AAC G |
|  | construct | DL_Pexp_055790_N_F | ATA CGA CAA ACG ACT TCA CGT CGG |
|  |  | DL_PEXP_055790_3N_R | GAG TAA AGA CTT GGG CTA GCA AGG |
|  | confirmation | DL_PEXP_055790_5N_F | CGG GTT ATG GAA CAA TTC CTC AGC |
|  |  | DL_Pexp_055790_3N_R | AGA GTT GGT ATG GGT GTT GAA GTT CG |
|  |  |  |  |
|  | 5' flank | DL_PEXP_096890_5_F | GTC AGC ACC AGA ATT CTA TTG TGG C |
|  |  | DL_PEXP_096890_5_R | CAC AGT GGA GGA CAT ACC CGT AAT TTT CTG TAT TGC TGG TTT TGT GAT ATC TGT CCG |
|  | pyrG | DL_Pexp_AfumiPyrG_F | CAG AAA ATT ACG GGT ATG TCC TCC AC |
|  |  | DL_Pexp_AfumiPyrG_R | TCA TGA CTT GCC GCA TAC TCT GG |
|  | 3' flank | DL_PEXP_096890_3_F | CTT ATA TGG CCA GAG TAT GCG GCA AGT CAT GAT GTC ATC CAC TTA GCC GGT CTG G |
| ∆*hfbE* (PEXP_096890) |  | DL_PEXP_096890_3_R | GAC CTG ATG CCA CAA AAC CGA TCC |
|  | construct | DL_PEXP_096890_N_F | CAT AGG GTT GTA GAC TTG GTA GGC G |
|  |  | DL_PEXP_096890_N_R | TCA ACT CAT TCC CCA CTA CTC ATG C |
|  | confirmation | DL_PEXP_096890_ORF_F | CTC TGT CTC AGC TTC ACC TAC C |
|  |  | DL_PEXP_096890_ORF_R | CAG CCT GGC TCT CTG GTA TAT CC |
|  |  |  |  |
|  | 5' flank | DL_PEXP_043320_5_F | TGC GGG AGT CTG AAT CTT TGC G |
|  |  | DL_PEXP_043320_5_R | CAC AGT GGA GGA CAT ACC CGT AAT TTT CTG TAT GGC GAT GGT GAT ATG GTA GCT GG |
|  | pyrG | DL_Pexp_AfumiPyrG_F | CAG AAA ATT ACG GGT ATG TCC TCC AC |
|  |  | DL_Pexp_AfumiPyrG_R | TCA TGA CTT GCC GCA TAC TCT GG |
|  | 3' flank | DL_PEXP_043320_3_F | TAT ATG GCC AGA GTA TGC GGC AAG TCA TGA AGT GAC GAA GGG TAC AAA GAG GAG G |
| ∆*hfbF* (PEXP_043320) |  | DL_PEXP_043320_3_R | TCT AAG TCC TGT CTT AGG ACA TCG C |
|  | construct | DL_PEXP_043320_N_F | GAA TGT AGA CAG AGC GTT CCA TAT CG |
|  |  | DL_PEXP_043320_N_R | TTG TTA GAT GCC CTG AGA AGA CTT GG |
|  | confirmation | DL_PEXP_043320_ORF_F | AAA GAT CAA CAT GCA CTG TAA CTT CGC |
|  |  | DL_PEXP_043320_ORF_R | GCG CCC TGA GAA AAG TAG AAT AAA CC |
|  |  |  |  |
|  | 5' flank | DL_PEXP_098360_5_F | CAA AGT GCA CCG CCA CAG ATG C |
|  |  | DL_PEXP_098360_5_R | CAC AGT GGA GGA CAT ACC CGT AAT TTT CTG TTT GAT GGA GTT TCC GAG GTG TTT GAA G |
|  | pyrG | DL_Pexp_AfumiPyrG_F | CAG AAA ATT ACG GGT ATG TCC TCC AC |
|  |  | DL_Pexp_AfumiPyrG_R | TCA TGA CTT GCC GCA TAC TCT GG |
|  | 3' flank | DL_PEXP_098360_3_F | GGC CAG AGT ATG CGG CAA GTC ATG AGT CTC GTT TGT TGA AAC TTT TAA CGG ATA TAT ATG |
| ∆*hfbG* (PEXP_098360) |  | DL_PEXP_098360_3_R | TCG AGG GAG TCG ATA CTT ACA ATG C |
|  | construct | DL_PEXP_098360_N_F | CTG CGC CGA GAT CGA AGT TTA CC |
|  |  | DL_PEXP_098360_N_R | TTC AGT ATT TCG TCT TTC ACG CGC C |
|  | confirmation | DL_PEXP_098360_ORF_F | ATC CTC TCC GTC TTC TCT CTT GTC G |
|  |  | DL_PEXP_098360_ORF_R | GTC TGT GTT GGA GAT TGT CAC TTA CC |
|  |  |  |  |
|  | 5' flank | DL_PEXP_096890_5_F | GTC AGC ACC AGA ATT CTA TTG TGG C |
|  |  | DL_Pexp_096890_hyg_5 R | CTC TAT TGA CCT ATA GGA CCT GAG TGA TGC TAT TGC TGG TTT TGT GAT ATC TGT CCG |
|  | hyg | DL_Pexp_hyg_F | GCA TCA CTC AGG TCC TAT AGG TCA ATA |
|  |  | DL_Pexp_hyg_R | GCA CTA GAT GGA CCA TAT TAT GCT CAA C |
|  | 3' flank | DL_Pexp_096890_hyg_3_F | AAG TTG AGC ATA ATA TGG TCC ATC TAG TGC TGT CAT CCA CTT AGC CGG TCT GG |
| ∆*hfbFE* |  | DL_PEXP_096890_3_R | GAC CTG ATG CCA CAA AAC CGA TCC |
|  | construct | DL_PEXP_096890_N_F | CAT AGG GTT GTA GAC TTG GTA GGC G |
|  |  | DL_PEXP_096890_N_R | TCA ACT CAT TCC CCA CTA CTC ATG C |
|  | confirmation | DL_PEXP_096890_ORF_F | CTC TGT CTC AGC TTC ACC TAC C |
|  |  | DL_PEXP_096890_ORF_R | CAG CCT GGC TCT CTG GTA TAT CC |
|  |  |  |  |
|  | 5' flank | DL_PEXP_098360_5_F | CAA AGT GCA CCG CCA CAG ATG C |
|  |  | DL_Pexp_triple_098360hyg_5_R | TACTCTATTGACCTATAGGACCTGAGTGATGCTTTGATGGAGTTTCCGAGGTGTTTGAAG |
|  | hyg | DL_Pexp_hyg_F | GCA TCA CTC AGG TCC TAT AGG TCA ATA |
|  |  | DL_Pexp_hyg_R | GCA CTA GAT GGA CCA TAT TAT GCT CAA C |
|  | 3' flank | DL_Pexp_triple_098360hyg_3_F | GAGCATAATATGGTCCATCTAGTGCGTCTCGTTTGTTGAAACTTTTAACGGATATATATG |
| ∆*hfbFEG* |  | DL_PEXP_098360_3_R | TCG AGG GAG TCG ATA CTT ACA ATG C |
|  | construct | DL_PEXP_098360_N_F | CTG CGC CGA GAT CGA AGT TTA CC |
|  |  | DL_PEXP_098360_N_R | TTC AGT ATT TCG TCT TTC ACG CGC C |
|  | confirmation | DL_PEXP_098360_ORF_F | ATC CTC TCC GTC TTC TCT CTT GTC G |
|  |  | DL_PEXP_098360_ORF_R | GTC TGT GTT GGA GAT TGT CAC TTA CC |
|  |  |  |  |
|  | 5' flank | DL_PEXP_071760_5_F | TCG GCT GTG CCT TAT ATT CAA GAC C |
|  |  | DL_Pexp_Quad_071760_hyg_5_R | CTC TAT TGA CCT ATA GGA CCT GAG TGA TGC TTT GAT AGA GGC TTT AGA GAA GGC GG |
|  | hyg | DL_Pexp_hyg_F | GCA TCA CTC AGG TCC TAT AGG TCA ATA |
|  |  | DL_Pexp_hyg_R | GCA CTA GAT GGA CCA TAT TAT GCT CAA C |
|  | 3' flank | DL_Pexp_Quad_071760_hyg_3_F | AAG TTG AGC ATA ATA TGG TCC ATC TAG TGC ATT GCA TTT CAT TGA GTG CAG CCT TTT CCG |
| ∆*hfbFEGC* |  | DL_PEXP_071760_3_R | GTA TCA AGG GAA CCG TAA AGC TTC C |
|  | construct | DL_PEXP_071760_N_F | GGA TAT TCG ACG CCA TGC ATA TAC TGA |
|  |  | DL_PEXP_071760_N_R | TGG GGT TTA TTC AGG TTC TTC TCG C |
|  | confirmation | DL_PEXP_071760_ORF_F | CTT TTC GCT GCT ACT GCC ATG G |
|  |  | DL_PEXP_071760_ORF_R | ATC ATT TTG GGG GAG CAC ATA CAG C |
|  |  |  |  |
|  | 5' flank | DL_PEXP_020490_5F | GCA TGT TGG TGG AAT AAT GTC TGT GG |
|  |  | DL-Pexp_Quint_020490_hyg_5_R | TCT ATT GAC CTA TAG GAC CTG AGT GAT GCC TTG AAG GTA TGG TAT TGA AAG ATT AAA ATC |
|  | hyg | DL_Pexp_hyg_F | GCA TCA CTC AGG TCC TAT AGG TCA ATA |
|  |  | DL_Pexp_hyg_R | GCA CTA GAT GGA CCA TAT TAT GCT CAA C |
|  | 3' flank | DL_Pexp_Quint_020490_hyg_3_F | AAG TTG AGC ATA ATA TGG TCC ATC TAG TGC GCT CAT CGC TTT GCA GTC AGT C |
| ∆*hfbFEGCB* |  | DL_PEXP_020490_3_R | TAC TTA GTC GCC CGT ATA TGT AGC |
|  | construct | DL_PEXP_020490_N_F | ACA CTA ACA TCC ACT TCC TGT GAA GC |
|  |  | DL_PEXP_020490_N_R | AGG ATT CAC CAA CTG GAC TTT CAC G |
|  | confirmation | DL_PEXP_020490_ORF_F | ATG CAG TTC ACT CTC TCC GCC |
|  |  | DL_PEXP_020490_ORF_R | GCG TTG AAA GGT GAT GGG TGC |
|  |  |  |  |
|  | 5' flank | DL_PEXP_055790_5_F | TCT ATA AAA AGC CCA ATG CCC ACG C |
|  |  | DL_SEXTU_PEXP_055790_5hyg_R | TAT TGA CCT ATA GGA CCT GAG TGA TGC GGT TGA TTG ATT TGA AAA GAT TGT TCA AAG TTG |
|  | hyg | DL_Pexp_hyg_F | GCA TCA CTC AGG TCC TAT AGG TCA ATA |
|  |  | DL_Pexp_hyg_R | GCA CTA GAT GGA CCA TAT TAT GCT CAA C |
|  | 3' flank | DL_SEXTU_PEXP_055790_3hyg_F | AAG TTG AGC ATA ATA TGG TCC ATC TAG TGC TGA AAA TGT CCA TCT GCA ATG AAA GCC |
| ∆*hfbFEGCBD* |  | DL_Pexp_055790_3_R | ATC GTT GTT GTC CGG AGG AAC G |
|  | construct | DL_Pexp_055790_N_F | ATA CGA CAA ACG ACT TCA CGT CGG |
|  |  | DL_PEXP_055790_3N_R | GAG TAA AGA CTT GGG CTA GCA AGG |
|  | confirmation | DL_PEXP_055790_5N_F | CGG GTT ATG GAA CAA TTC CTC AGC |
|  |  | DL_Pexp_055790_3N_R | AGA GTT GGT ATG GGT GTT GAA GTT CG |
|  |  |  |  |
|  | 5' flank | DL_Pexp_062290_5_F | CAC CCT CAC TGT TTT AGT GGG ACG |
|  |  | DL_Pexp_Sept_Pexp_062290_5_R | CTC TAT TGA CCT ATA GGA CCT GAG TGA TGC TGT GAT GGT TTG GAT TTG AAC GAG AGG A |
|  | hyg | DL_Pexp_hyg_F | GCA TCA CTC AGG TCC TAT AGG TCA ATA |
|  |  | DL_Pexp_hyg_R | GCA CTA GAT GGA CCA TAT TAT GCT CAA C |
|  | 3' flank | DL_Pexp_Sept_Pexp_062290_3_F | AAG TTG AGC ATA ATA TGG TCC ATC TAG TGC TTA GAT GTC CTG TTC ATT TCA TAT CTC GTG |
| ∆*hfbFEGCBDA* |  | DL_Pexp_062290_3_R | TAT CTC CAG CCC AGA ATT GAA TGC |
|  | construct | DL_Pexp_062290_5N_F | GTA ATG CGG ACT ATG TAT AGA CCA AGG |
|  |  | DL_Pexp_062290_3N_R | GAT GCC AAT ATA CCC TCC AAC CAG C |
|  | confirmation | DL_Pexp_062290_ORF_F | GAC CAG GCT CAG CTC TCT TGC |
|  |  | DL_Pexp_062290_ORF_R | GGA GTT CTG TGA TGT GTT AGT TGA GC |

Table S1B. **List of primers used in reverse transcriptase PCR**

| **Gene** | **Primer ID** | **Primer Sequence** | **cDNA size (bp)** | **gDNA size (bp)** |
| --- | --- | --- | --- | --- |
| *hfbA* (PEXP_062290) | PEXP_062290_RT_F | GAC CAG GCT CAG CTC TCT TGC | 163 | 163bp (no intron) |
|  | PEXP_062290_RT_R | CCT GGA GAT CCA GCT TGG AGC |  |  |
|  |  |  |  |  |
| *hfbB (*PEXP_020490) | DL_PEXP_020490_RT_F | CTT ATG CCG GTG ACA CCA CCG | 153 | 206 |
|  | DL_PEXP_020490_RT_R | TGG GGA TAC CGA TGA CGA TAG GG |  |  |
|  |  |  |  |  |
| *hfbC* (PEXP_071760) | DL_PEXP_071760_RT_F | TGA GGG TCT CGG TCT TGG CC | 132 | 185 |
|  | DL_PEXP_071760_RT_R | GAG TTG GAG TTC TGG CAG CAG G |  |  |
|  |  |  |  |  |
| *hfbD* (PEXP_055790) | PEXP_055790_RT_F | AAA TAC TGC AAC GGT GGC ACA TTC C | 139 | 193 |
|  | PEXP_055790_RT_R | TCC TGT AAC CAA AAG CAA CCT CTG C |  |  |
|  |  |  |  |  |
| *hfbE* (PEXP_096890) | DL_PEXP_096890_RT_F | GCA AGG TTA TAC CCC CAT GTG TTG C | 131 | 217 |
|  | DL_PEXP_096890_RT_R | CAG CCT GGC TCT CTG GTA TAT CC |  |  |
|  |  |  |  |  |
| *hfbF* (PEXP_043320) | DL_PEXP_043320_RT_F | GTG ATC AAT TGG ACC AGC CTC AGC | 112 | 178 |
|  | DL_PEXP_043320_RT_R | TCA GCA TCT TGG AAG ATA ACG TGG C |  |  |
|  |  |  |  |  |
| *hfbG* (PEXP_098360) | DL_PEXP_098360_RT_F | TTG TCG ACC CCC TCC TGT TGG | 133 | 195 |
|  | DL_PEXP_098360_RT_R | AGG AGA TTA GCC TCG GTG CAG C |  |  |
|  |  |  |  |  |
| Actin | DL_PEXP_ACTIN_RT_F | ATC CAC GTC ACC ACC TTC AAC TCC | 110 | 175 |
|  | DL_PEXP_ACTIN_RT_R | GAG ATA CCG GGG TAC ATG GTG G |  |  |

Table S1C. ***P. expansum* strains used in this study and their genotypes.**

| **Strain Name** | **Brief Genotype** | **Genotype** | **Strain Source** |
| --- | --- | --- | --- |
| TWW 13.1 | Control (WT) | Control strain Δ*ku70*::sixΔ*pyrG*::six, *AfumipyrG@ku70 locus* | Wang *et al*., 2021 |
| TDL 21.3 | *∆hfbB* | ∆*pexp*_020490::*A. fumigatus pyrG* Δ*ku70*::*six* | This study |
| TDL 17.2 | *∆hfbF* | ∆pexp_043320::*A. fumigatus pyrG* Δ*ku70*::*six* | This study |
| TDL 18.2 | *∆hfbC* | ∆*pexp*_071760::*A. fumigatus pyrG* Δ*ku70*::*six* | This study |
| TDL 19.2 | *∆hfbE* | ∆pexp_096890::*A. fumigatus pyrG* Δ*ku70*::*six* | This study |
| TDL 20.3 | *∆hfbG* | ∆*pexp*_098360::*A. fumigatus pyrG* Δku70::six | This study |
| TDL 27. 1 | *∆hfbD* | ∆pexp_055790::*A. fumigatus pyrG* Δku70::six | This study |
| TDL 28.1 | *∆hfbA* | ∆*pexp_062290*::*A. fumigatus pyrG* Δku70::six | This study |
| TDL 24.1 | *∆hfbFE* | ∆*pexp_043320*::*A. fumigatus pyrG* ∆pexp_096890::*six* Δ*ku70*::*six* | This study |
| TDL 26.2 | *∆hfbFEG* | ∆pexp_043320::*A. fumigatus pyrG* ∆*pexp*_096890::*six* ∆*pexp*_098360::*six* Δ*ku70*::*six* | This study |
| TDL 30.1 | *∆hfbFEGC* | ∆pexp_043320::*A. fumigatus pyrG* ∆*pexp*_096890::*six* ∆*pexp*_098360::*six* ∆*pexp*_071760::*six* Δ*ku70*::*six* | This study |
| TDL 31.1 | *∆hfbFEGCB* | ∆*pexp*_043320 ∆*pexp*_096890::*six* ∆*pexp*_098360::*six* ∆*pexp*_071760::*six* ∆*pexp*_02490::*six-𝛃-rec-hpt-six* Δ*ku70*::*six* |  |
| TDL 32.1 | *∆hfbFEGCB* | ∆*pexp*_043320 ∆*pexp*_096890::*six* ∆*pexp*_098360::*six* ∆*pexp*_071760::*six* ∆*pexp*_02490::*six* Δ*ku70*::*six* | This study |
| TDL 34.1 | *∆hfbFEGCBD* | ∆*pexp*_043320 ∆*pexp*_096890::*six* ∆*pexp*_098360::*six* ∆*pexp*_071760::*six* ∆*pexp*_02490::*six* ∆*pexp*_055790::*six* Δ*ku70*::*six* | This study |
| TDL 35.1 | *∆hfbFEGCBDA* | ∆pexp_043320 ∆*pexp*_096890::*six* ∆*pexp*_098360::*six* ∆*pexp*_071760 ::*six* ∆*pexp*_02490::*six* ∆*pexp*_055790::*six* ∆*pexp*_062290::*six-𝛃-rec-hpt-six* Δ*ku70*::*six* | This study |
| TDL 36.1 | *∆hfbFEGCBDA* | ∆pexp_043320 ∆*pexp*_096890::*six* ∆*pexp*_098360::*six* ∆*pexp*_071760 ::*six* ∆*pexp*_02490::*six* ∆*pexp*_055790::*six* ∆*pexp*_062290::*six* Δ*ku70*::*six* | This study |
